# Poly(ADP-Ribose) Polymerase 1 (PARP-1) Regulates Ribosomal Biogenesis in *Drosophila* Nucleoli

**DOI:** 10.1371/journal.pgen.1002442

**Published:** 2012-01-05

**Authors:** Ernest K. Boamah, Elena Kotova, Mikael Garabedian, Michael Jarnik, Alexei V. Tulin

**Affiliations:** Fox Chase Cancer Center, Philadelphia, Pennsylvania, United States of America; Cornell University, United States of America

## Abstract

Poly(ADP-ribose) polymerase 1 (PARP1), a nuclear protein, utilizes NAD to synthesize poly(AD-Pribose) (pADPr), resulting in both automodification and the modification of acceptor proteins. Substantial amounts of PARP1 and pADPr (up to 50%) are localized to the nucleolus, a subnuclear organelle known as a region for ribosome biogenesis and maturation. At present, the functional significance of PARP1 protein inside the nucleolus remains unclear. Using PARP1 mutants, we investigated the function of PARP1, pADPr, and PARP1-interacting proteins in the maintenance of nucleolus structure and functions. Our analysis shows that disruption of PARP1 enzymatic activity caused nucleolar disintegration and aberrant localization of nucleolar-specific proteins. Additionally, PARP1 mutants have increased accumulation of rRNA intermediates and a decrease in ribosome levels. Together, our data suggests that PARP1 enzymatic activity is required for targeting nucleolar proteins to the proximity of precursor rRNA; hence, PARP1 controls precursor rRNA processing, post-transcriptional modification, and pre-ribosome assembly. Based on these findings, we propose a model that explains how PARP1 activity impacts nucleolar functions and, consequently, ribosomal biogenesis.

## Author Summary

Ribosome assembly happens primarily in the subnuclear organelle nucleolus. In the nucleolus, ribosomes are assembled into a multmeric complex, composed of rRNA and ribosomal proteins. Although a lot is known about ribosomes and how they function, very little is known about the mechanism that facilitates the assembly of these multimeric protein complexes in the nucleolus. Here, we provide evidence that a nuclear protein, PARP1, primarily known for its DNA damage repair and transcriptional activities, also plays a critical role in the assembly of ribosomes. Using the *Drosophila* model system, we show that PARP1 localization within the nucleolus impacts such nucleolar activities as rRNA processing and ribosome biogenesis. We show that, when PARP1 activity is disrupted, nucleolar proteins that normally co-localize under wild-type conditions disperse into the nucleoplasm and do not show any co-localization. We also show that some nucleolar proteins, essential for rRNA processing, also interact with pADPr, which keeps these proteins close to precursor rRNA. When PARP1 activity was disrupted, we observed precursors rRNA accumulation and a concomitant decrease in the levels of ribosomes. Together, our data suggest a novel activity for PARP1 and highlight a potential mechanism associated with ribosome biogenesis in the nucleolus.

## Introduction

The nuclear substructure, nucleolus, is a site commonly associated with translational complex assembly, and thus functions as a major regulator of cell growth [1]. The nucleolus is composed of an array of tandem repeated units of ribosomal RNA (rRNA) genes, some of which are transcribed, while others remain in an inactive heterochromatic state [2]–[4]. Additionally, the nucleolus contains a diverse pool of proteins, most of which are involved primarily with transcription, processing, and modification of rRNA transcripts, ribosome assembly, and transport of translational competent ribosome to the cytoplasm [1], [5]. Actively growing yeast cells produce about 2000 ribosomes per minute, underscoring the amount of metabolic investment made by a cell during growth towards ribosome production [6]. Ample data also suggest that the regulation of rRNA synthesis and production of ribosomes can influence cancer progression [7]. However, despite the advances in nucleolar research, the sequence of molecular events that coordinates ribosomal biogenesis with cell growth, especially in highly proliferative cells, such as cancer cells, is poorly understood.

PARP1 protein, utilizes NAD as a substrate to generate poly(ADP-ribose) (pADPr) for automodification and the modification of acceptor proteins, such as chromatin-associated histone proteins [8]–[12]. Glutamate residues of acceptor proteins serve as sites for poly(ADP-ribose) attachment [13]. Modification of proteins by PARP1 alters their localization in the cell and modifies their biological activities [14]–[17]. Since automodification disrupts the physiological activity of PARP1, it is necessary to counteract the addition of ADPr polymers. Thus, to maintain active PARP1 protein levels, ADPr polymers are removed and subsequently metabolized by PARG [18]–[21]. PARG knockout results in the accumulation of automodified PARP1, which is rendered incapable of re-associating with DNA or further catalyzing ADPr [20], [22].

*Drosophila* nucleoli contain large quantities of PARP1 and pADPr, and display considerable amounts of PARP1 activity [23], [24]. Whereas nucleoli structure disintegrates completely in *Parp1* mutants, the ectopic expression of PARP1 cDNA restores proper assembly of nucleolar components and structure [23]. Although PARP1 does not contain any known nucleolar localization signal, it has been proposed that PARP1 localization in the nucleolus appears to depend on nucleolar activity because a large amount of PARP1 translocates from the nucleolus when ribosomal DNA (rDNA) transcription is inhibited [25], [26]. Nucleolar components, such as Fibrillarin [20], Nucleolin, and Nucleoplasmin/B23 [26], [27], interact and colocalize with PARP1 in the nucleus and undergo modification by pADPr [28]. In addition, a number of ribosomal proteins have been shown to interact with PARP1 protein [29], [30]. Both the nucleolar localization and interaction with nucleolar proteins suggest that PARP1 may function in regulating some aspect of nucleolar activity. Here we evaluate the roles of PARP1, ADPr, and nucleolar proteins that interact with PARP1 to determine the impact of PARP1 in regulating nucleolar structure and functions.

## Results

### Disruption of PARP1 activity causes improper localization of nucleolar-specific proteins

**Figure 1:**
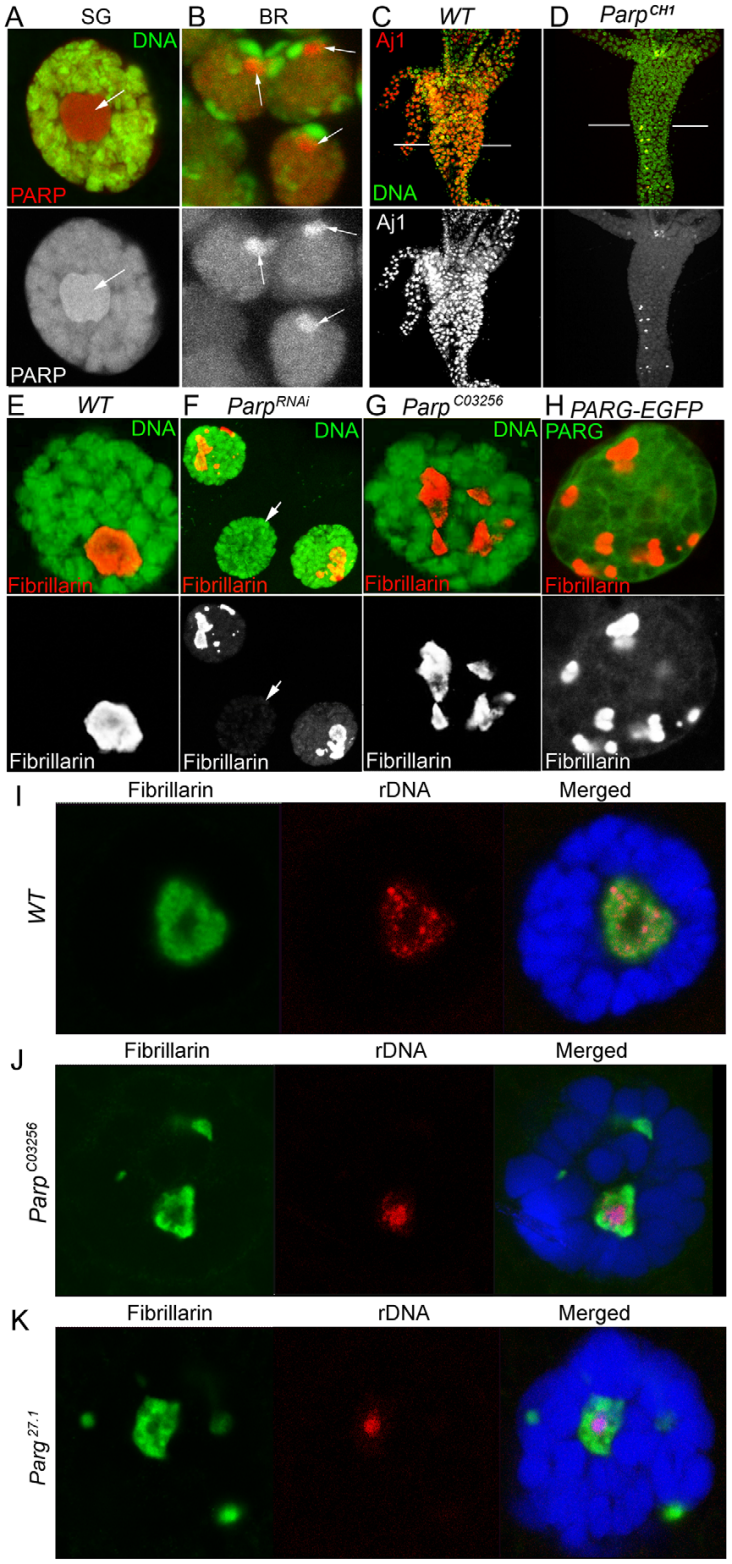
PARP1 controls nucleolar structural integrity. A–B. PARP1 protein localizes to nucleoli in all *Drosophila* tissues, including polytene nuclei of larval salivary glands (A) and diploid nuclei of larval brain (B). The dissected larval salivary glands and brains expressing PARP1-DsRed (red) were stained with the DNA binding dye Draq5 (green). Positions of nucleoli are indicated with arrows. SG – larval salivary gland; BR – larval brain. C–D. PARP1 deletion displaces nucleoli protein as detected by nucleoli-specific antibody AJ1 (red). AJ1 detects nucleoli in every cell of *Drosophila* midintestine in wild-type second-instar larvae (C), but only in a few cells in *Parp^CH1^* mutants (D). DNA is detected with OliGreen dye (green). E–H. Deletion or disruption of PARP1 protein functions disintegrates nucleolus structure. Salivary glands from wild-type (E), *Parp^RNAi^* expressing (F), hypomorphic *Parp* mutant, *Parp^C03256^* (G), and overexpressing antagonist of PARP1, PARG (H) 3rd instar larvae were stained for the nucleolar specific protein Fibrillarin (red). In wild-type cells, Fibrillarin (red) localizes in an intact single nucleolus (E). Compromising PARP1 protein activity (F–H) causes nucleolus fragmentation, as indicated by the aberrant localization of Fibrillarin (red). DNA is detected with Draq5 dye (green) (E–G). Overexpression of PARG-EGFP protein (H) is detected by EGFP autofluorescence (green). Arrow (F) indicates the nucleus of *Parp^RNAi^*-expressing larval salivary gland that shows undetectable levels of Fibrillarin protein. White bars of (F) indicate areas that were subjected to TEM analysis shown in Figure 6A–6D. I–K. Nucleolar fragments in *Parp^C03256^* and *Parg^27.1^* do not contain rDNA. Dissected salivary glands from wild-type (I) *Parp^C03256^* (J) and *Parg^27.1^* (K) 3rd instar larvae were hybridized with rDNA probe (red) and stained for the Fibrillarin protein (green). DNA was detected with Draq5 dye (blue).

We previously reported that *Drosophila* PARP1 is broadly distributed on chromosome and is enriched in active chromatin [24]. In all tissues of wild-type *Drosophila*, nucleoli contained large quantities of PARP1 and pADPr, and display considerable amounts of PARP1 activity [23]. We determined that more than 40% of nuclear PARP1 (Figure 1A and 1B) and pADPr (Figure S1) are localized within the nucleolus. Since PARP1 activity within the nucleolus is not clearly defined, we investigated how its enzymatic activity affects nucleolar architecture and functions. Here we show that depletion of PARP1 protein in *Parp^CH1^* mutant results in mis-localization of nucleolar specific proteins in all *Drosophila* tissues analyzed (Figure 1C and 1D). Although a large portion of nucleolar proteins shifted their localization to the cytoplasm, the total amount of these proteins did not change (Figure S2). This finding suggests that PARP1 may function in part to control nucleolar structural integrity by maintaining proteins within this subnuclear structure. To address this, we analyzed the localization of nucleolar-specific proteins under different PARP1 genetic backgrounds. We used Fibrillarin, a nucleolar protein involved in rRNA processing and maturation, as a marker for nucleolar integrity [31]. Consistent with our finding regarding *Parp^CH1^* mutants [23], disruption of PARP1 activity by expressing *Parp^RNAi^* (Figure S3), using hypomorphic mutants *Parp^C03256^* [32], or overproducing the antagonist of PARP1, PARG protein, caused nucleolar fragmentation, as detected by anti-Fibrillarin antibody (Figure 1E–1H). Whereas these additional nucleolus-like structures do not contain rDNA (Figure 1I–1K), they cannot take part in the ordered process of ribosomal biogenesis. Our data shows that PARP1 protein is required for nucleolar structural integrity and compromising the enzymatic activity of the protein always leads to nucleolar disruption. This finding suggests that the product of PARP1 enzymatic reaction, poly(ADP-ribose) (pADPr), may be an important component of the nucleolus and that it may serve as a matrix for nucleolar protein binding, keeping them together in proximity to precursor rRNA.

**Figure 2:**
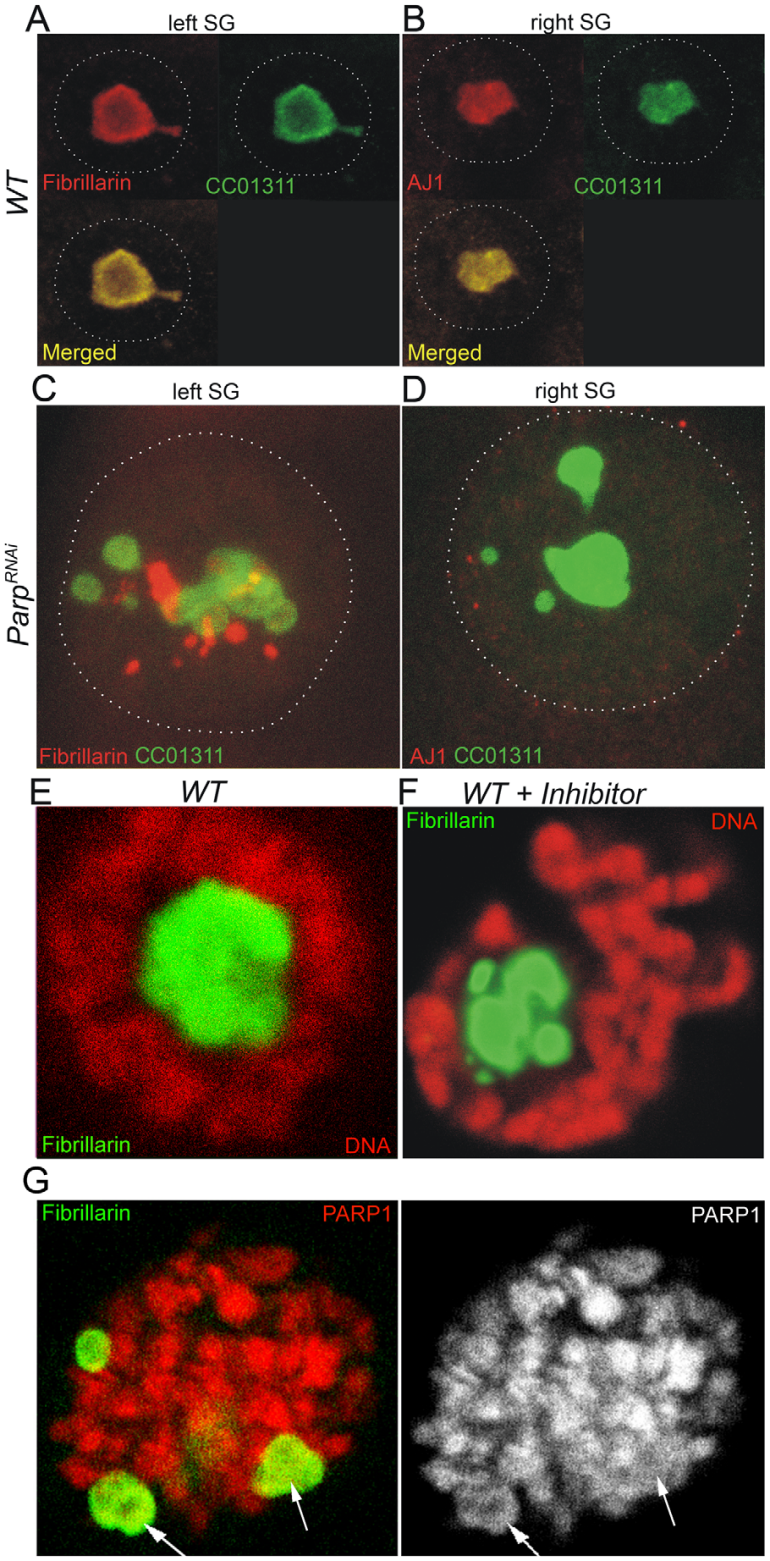
Compromising PARP1 enzymatic activity disrupts co-localization of nucleolar proteins. A–D. The dissected pairs of salivary glands expressing CC01311 nucleolar GFP were split into left and right individual glands and stained separately using antibody against nucleolar protein Fibrillarin (A, C) and nucleolar antibody AJ1 (B, D). Three nucleolar markers, Fibrillarin, CC01311, and AJ1 co-localize in wild-type third-instar larvae salivary gland nucleoli [53], but show completely different localization in *Parp^RNAi^*-expressing tissues (C–D). Nuclear membrane envelop is outlined. E–F. Chemical inhibition of PARP1 leads to immediate disruption of nucleolar domain. Wild-type third-instar larvae (E) were cultured 12 hours in the presence of a NAD analogue PARP1 inhibitor, 3-aminobenzamide (F). Nucleoli were detected by anti-Fibrillarin antibody (green). DNA was detected by Draq5 dye (red). The separation of a single nucleolar domain (E) into multiple “blobs” is clearly seen upon PARP1 inhibition (F). G. Prolonged treatment of third instar larvae with PARP1 inhibitor. Nucleoli were detected by anti-Fibrillarin antibody (green). PARP1 was detected by PARP1-DsRed autofluorescence (red). Arrows indicate nucleoli-like blobs.

To substantiate the role of pADPr in nucleolar structure and localization of nucleolar- specific proteins, we depleted *Parp1* using RNAi expression (Figure S3) and analyzed the resulting salivary gland for localization of three nucleolar-specific markers: Fibrillarin, AJ1, and nucleolar GFP-exon trap marker [33], CC01311 (Figure 2). In wild-type larvae, Fibrillarin, AJ1, and CC01311 localized in an intact single nucleolar structure (Figure 2A and 2B). However, RNAi depletion of *Parp1* caused nucleolar-specific proteins to be localized completely independent from each other, unlike in wild-type tissue (Figure 2C and 2D).

The observation that depletion of PARP1 protein affected the localization of nucleolar proteins caused us to determine whether PARP1 enzymatic activity, and not PARP1 protein itself, is essential for nucleolar integrity. We inhibited the activity of PARP1 by culturing third-instar larvae in the presence of the NAD analogue, 3 aminobenzamide (3AB). Upon inhibition of PARP1 activity by 3AB, we observed the disintegration of nucleoli, as indicated by fragmented Fibrillarin (Figure 2E and 2F). A prolonged treatment of *Drosophila* tissues with PARP1 inhibitor exacerbates nucleolus fragmentation but does not affect localization of PARP1 protein within these nucleolus-like fragments (Figure 2G). Together, these results support the hypothesis that PARP1 enzymatic activity and pADPr are required for maintaining nucleolar structure.

### Mutating PARG reveals differential localization and binding of nucleolar proteins to pADPr

**Figure 3:**
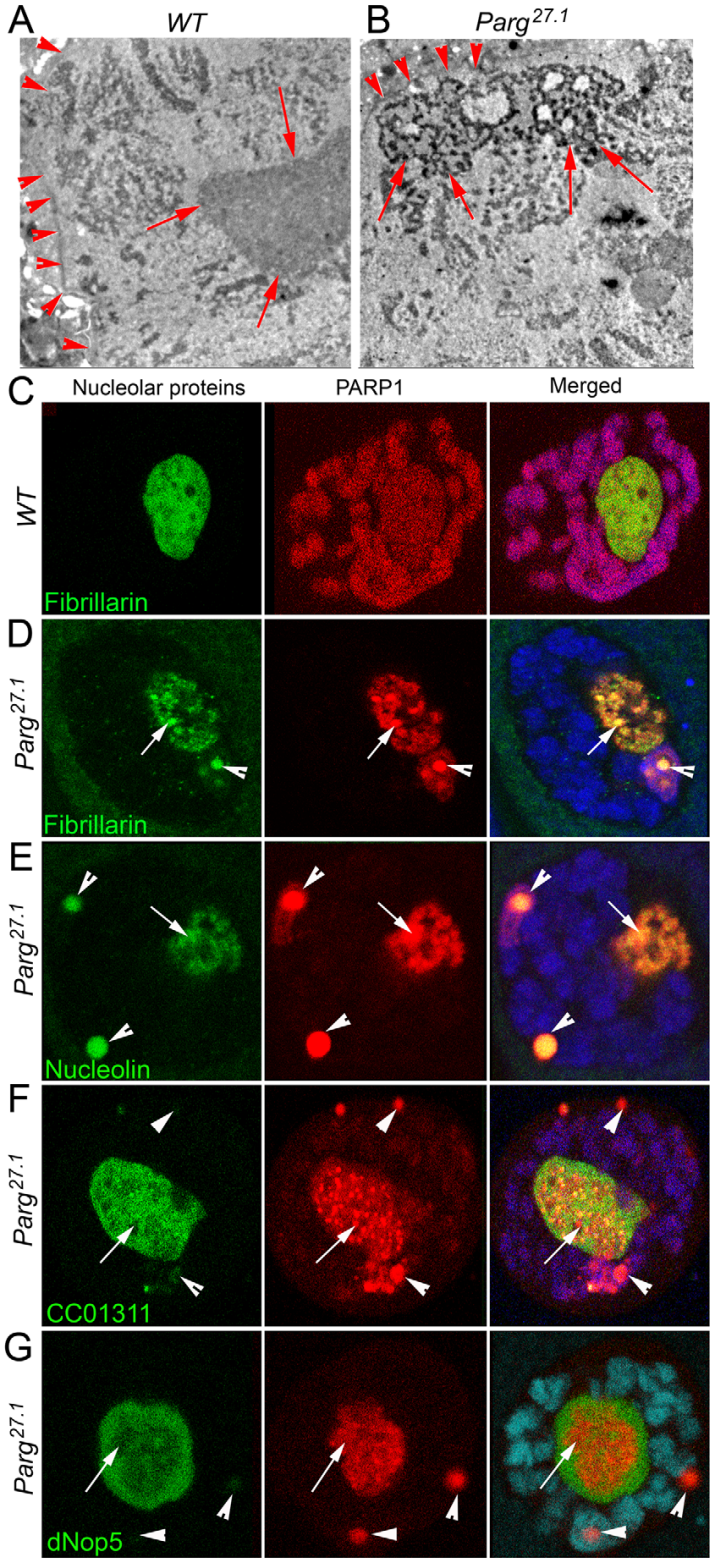
Mutating PARG disrupts nucleoli and reveals differential localization of nucleolar proteins. A–B. The structure of wild-type nucleolus detected by EM microscopy (A) is affected in *Parg^27.1^* mutants (B). In contrast to clear homogeneous (grey) content of wild-type nucleolus, *Parg^27.1^* mutant nucleoli accumulate “holes” and “aggregates” of proteins or chromatin (black). Arrows indicate the nucleolus. Arrowheads are pointing to the nuclear envelope. C. Nucleolar proteins colocalize with PARP1 (red) in wild-type nucleoli. Fibrillarin (green) protein is shown. D–G. Mutating PARG displaces PARP1 protein from chromatin to Cajal Bodies (arrowheads) and “traps” PARP1 within condensed nucleolar blocks (arrows). One class of nucleolar proteins completely co-localizes with PARP1 in CB and nucleoli (D–E). Another group of nucleolar proteins (F–G) behave independently from PARP1. This group of proteins has a homogeneous localization in nucleoli (arrows) and could barely be detected in CBs (arrowheads).

To further evaluate nucleoli structural integrity, we used Transmission Electron Microscopy (TEM) analysis to detect any nucleolar abnormalities associated with disrupting PARP1 activity. TEM revealed significant changes in nucleoli in both wild-type and *Parg^27.1^* mutant nuclei (Figure 3A and 3B). Wild-type nucleoli are typically located close to the middle part of nuclei and appear to be almost homogenic (Figure 3A). In contrast, nucleoli of *Parg^27.1^* mutants contain heavily condensed areas positioned close to nuclear lamina (Figure 3B). Such structural changes may impact nucleolar functions and affect the steady state equilibrium of ribosomal rRNA processing. Therefore, we tested localization of nucleolar proteins reported to participate in rRNA processing and maturation in wild-type and *Parg^27.1^* mutant nuclei using immunostaining.

In addition to Fibrillarin, we evaluated colocalization using Nucleolin and dNop5, both of which have been shown to be involved in nucleolar rRNA processing and ribosomal maturation [34], [35]. Additionally, we used Casein Kinase II α (CKIIα as a nucleolar marker, since previous reports have shown that it participates in rDNA transcription by phosphorylating components of RNA polymerase I [36]. Our results from this analysis shows that, in wild-type nucleoli, all tested nucleolar proteins demonstrate similar homogenic staining and co-localization with PARP1 protein (Figure 3C, only Fibrillarin protein is included). In contrast, nucleolar proteins in *Parg^27.1^* nuclei accumulate in two distinct locations. This suggests that, their exclusive localization within the nucleolus requires the activity of a functional PARP1 protein.

Previously, we demonstrated that in *Parg^27.1^* mutants, most of PARP1 protein is automodified, and, together with all other poly(ADP-ribosyl)ated proteins and pADPr binding proteins, it is either targeted to Cajal Bodies (CBs) or arrested inside abnormal condensed nucleolar substructures [20], [32]. Based on this observation, we proposed that pADPr modification would lead to co-localization with automodified PARP1 in CBs. One group of proteins, Fibrillarin and Nucleolin, co-localized with PARP1 inside condensed blocks of the nucleolus and in CBs (Figure 3D and 3E). However, another set of proteins, including nucleolar markers CC01311, dNop5 and CKIIα were preferentially antagonistic to PARP1 localization and did not accumulate in CBs (Figure 3F and 3G; Figure S4). These observations suggested a direct interaction of the first group of proteins with automodified PARP1. This interaction likely affects the location of these proteins in the nucleolus and thus may impact nucleolar activity. In contrast, CC01311, dNop5 and CKIIα do not interact with automodified PARP1. As a result, their positioning in the nucleolus is determined by PARP1-independent mechanisms. The existence of a selected group of nucleolar proteins requiring PARP1 enzymatic activity for their localization suggested to us that PARP1 may function in the nucleolus by binding these proteins through pADPr attachment.

**Figure 4:**
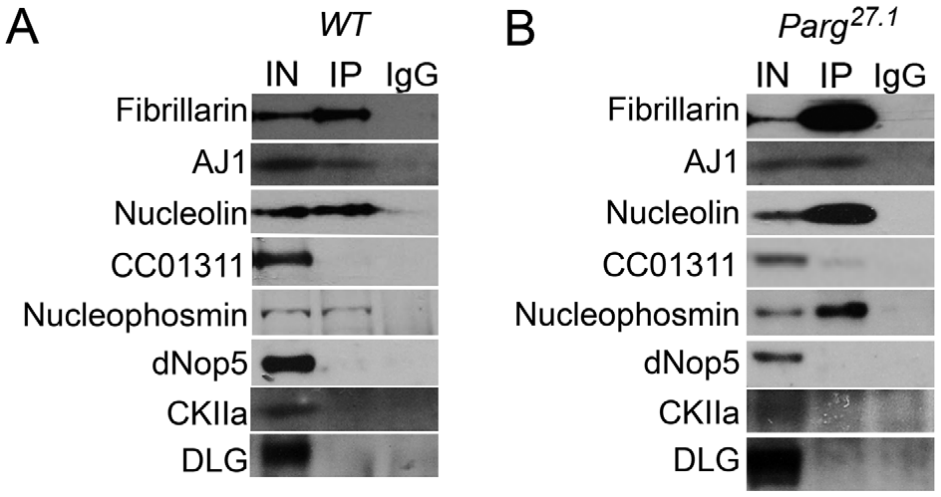
Nucleolar proteins show differential interaction with pADPr. Immunoprecipitation assays using mouse anti-pADPr antibody. Wild-type (A) and *Parg^27.1^* mutant (B) third-instar larvae were used. The following antibodies were used for Western blot analysis: rabbit anti-Fibrillarin; rabbit AJ1; rabbit anti-Nucleolin; rabbit anti-GFP (to detect nucleolar CC01311 marker); rabbit anti-Nucleophosmin; rabbit anti-dNop5; rabbit anti-CK2α; and mouse anti-Dlg (as a control).

To further test our hypothesis that a specific sub-group of nucleolar proteins preferentially interacts with pADPr, we performed co-immunoprecipitation experiments (Co-IP) using anti-pADPr antibody. Indeed, we found that Fibrillarin, AJ1, Nucleolin, and Nucleophosmin co-precipitate with pADPr in wild-type animals and *Parg^27.1^* mutants (Figure 4A and 4B), while other nucleolar proteins, including CC01311, dNop5 and CKIIα, do not show interaction with pADPr in wild-type nuclei (Figure 4A). Interestingly, a slight interaction of CC01311 protein with pADPr was detected in *Parg^27.1^* mutant (Figure 4B), although this weak interaction is likely indirect and may be mediated by other nucleolar components.

This result suggests that by binding a specific set of proteins to pADPr, PARP1 may determine the order of steps that occur during the process of ribosome biogenesis. Therefore, we further tested if mutating PARP1 or PARG affects any specific steps involved in ribosome production.

### Inhibition of PARP1 activity disrupts rRNA processing

**Figure 5:**
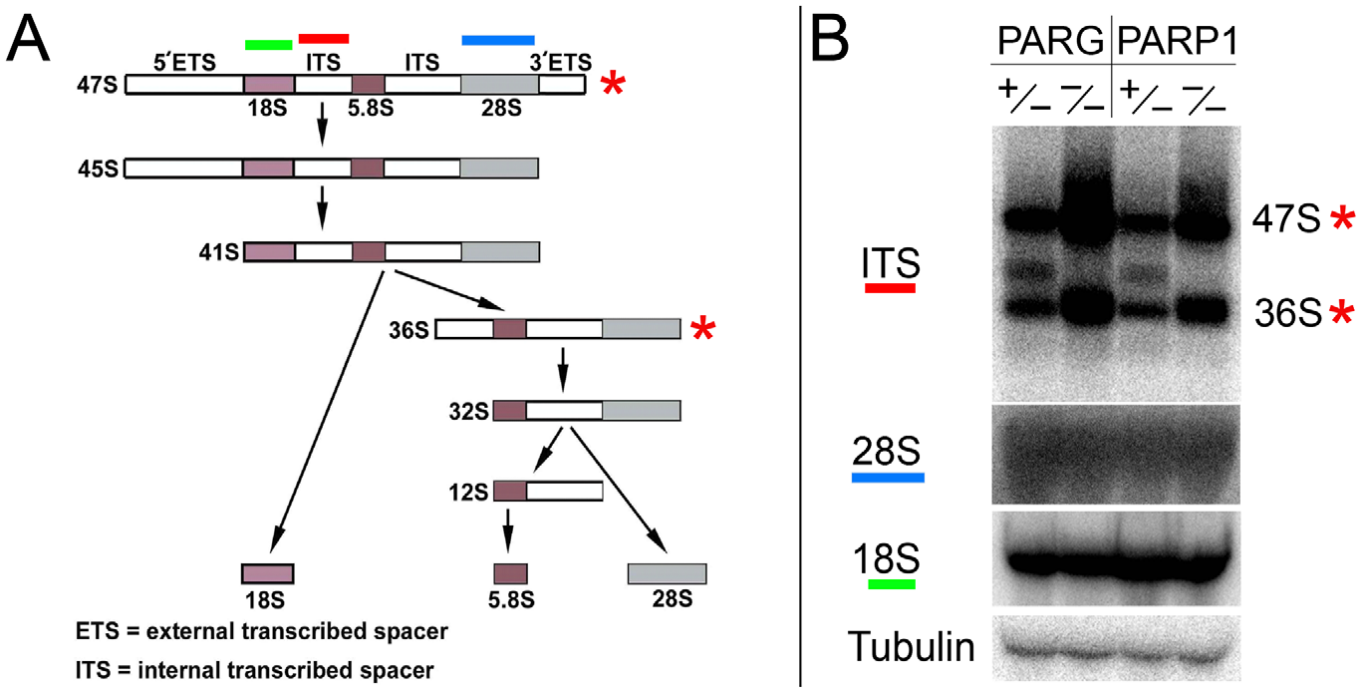
Production of rRNA intermediates increases upon disruption of PARP1 or PARG activity. A. Diagrammatic representation of mammalian rRNA transcript processing. The final 18S rRNA product becomes part of the 40S small ribosomal complex, while the 5.8S and 28S rRNA transcripts are incorporated into the large 60S ribosomal complex. Red bar above the scheme indicates internal transcriber spacer (ITS) probe, which was used to detect intermediates of rRNA processing on Northern blots. B. Northern blot analysis of rRNA intermediates. Disruption of PARG or PARP1 activity enhances the production of rRNA intermediates (right lanes) compared to the heterozygous (left lanes), which has normal PARP1 and PARG activity. The total level of mature rRNA does not increase (18S and 28S). Labeled probe to *Drosophila* Tubulin mRNA was used as a loading control.

Resident nucleolar proteins, such as Fibrillarin, have critical roles in rRNA processing, modification, and maturation [31], [37]–[39]. Our observation of fragmented localization of nucleolar-specific proteins, some of which are critical for rRNA maturation, prompted us to investigate the effect of inhibiting PARP1 activity on rRNA processing and maturation. The transcription of rDNA produces a precursor molecule which is subjected to multiple rounds of exonucleolytic and endonucleolytic cleavages by resident nucleolar protein complexes to generate mature 18S, 5.8S, and 28S rRNA final products [40], [41]. We reasoned that the displacement of nucleolar proteins involved in rRNA processing will have a disruptive effect on the generation of mature rRNA products. To evaluate our hypothesis, we isolated RNA from both wild-type and mutant larvae and analyzed precursor rRNA intermediates using Northern blot. Surprisingly, we found no quantifiable difference between mature rRNA amounts in wild-type, *Parg^27.1^*, and *Parp^C03256^* mutants (Figure 5, 18S and 28S). Instead, a significant increase in rRNA intermediates was detected in *Parg^27.1^* and *Parp^C03256^* mutants (Figure 5, Figure S5).

When taken together with the observations reported above, this suggest that displacement of key components of ribosomal biogenesis, which occurs in *Parg^27.1^* and *Parp^C03256^* mutants, results in rRNA processing delays and over-accumulation of immature intermediates. In contrast, the initial 47S rRNA precursor product and the intermediate 36S product were effectively processed in *Parg^27.1^* and *Parp^C03256^* heterozygotes, and accumulation of these two precursor products was observed in larvae lacking PARP1 or PARG function (Figure 5, red asterisks). The last finding indicates that mutating pADPr pathway does not affect the rate of rDNA transcription, but blocks specific steps of precursor rRNA maturation and accumulation of immature ribosomes. To test this hypothesis, we then proceeded to compare ribosomal content of wild-type, *Parp1*, and *Parg* mutants.

### PARP1 is required for ribosome biogenesis

Our observation of rRNA intermediates processing defects in *Parg^27.1^* and *Parp^C03256^* larvae caused us to investigate if any changes occur in the assembly of ribosomal subunit in these mutants. To assemble competent translational machinery, mature rRNA products form complexes with accessory proteins to form the small (40S) and large (60S) subunit particles [1], [5]. These particles are released into the nucleoplasm for further maturation and then exported into the cytoplasm where they become part of the translational machinery [1]. The activity and effectiveness of the translational machinery often can be monitored by presence of multiribosomal complexes on mRNAs, called polysomes [42].

**Figure 6:**
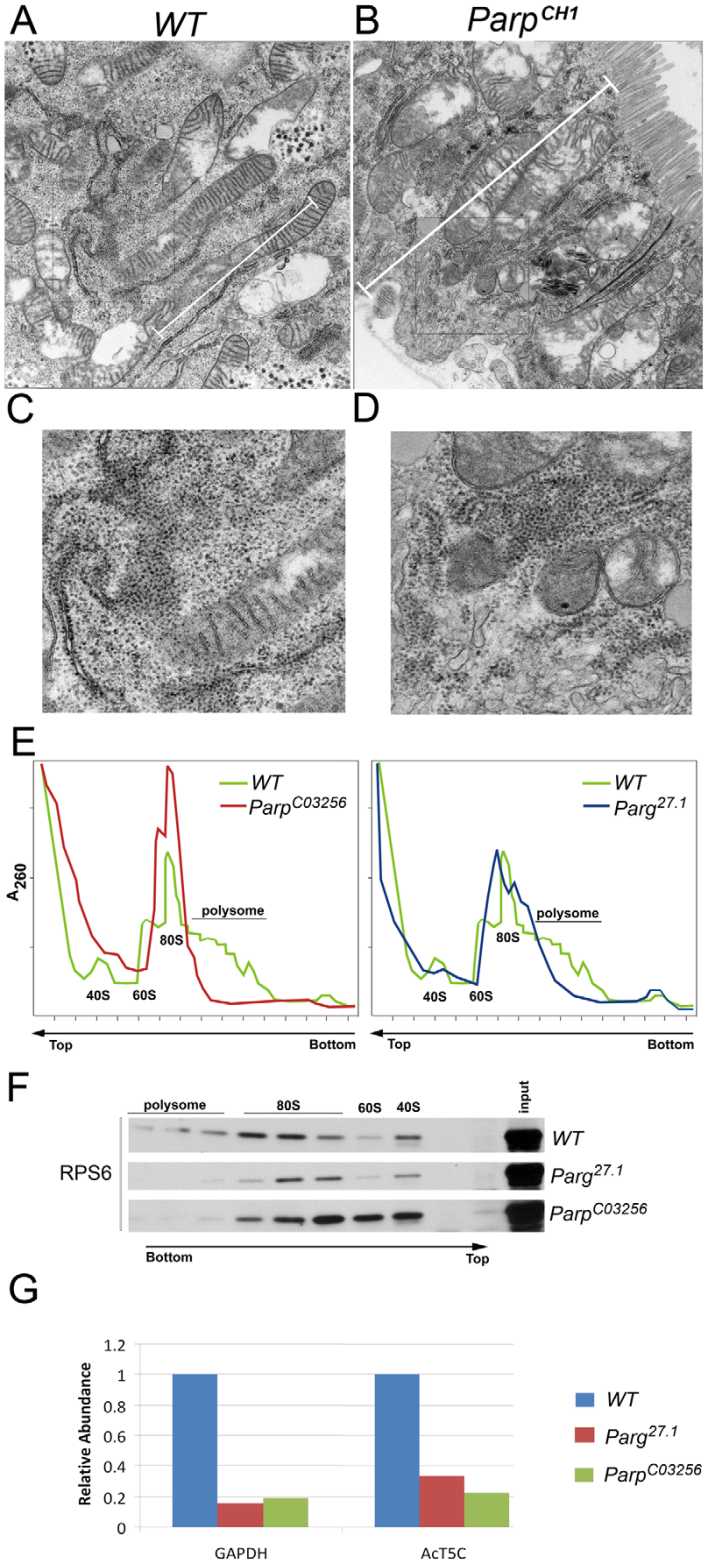
PARP1 is required for ribosomal biogenesis. A–D. TEM images of sections through midintestine of wild-type (A, C) and *Parp^CH1^* mutant second-instar larvae (B, D). Sections were made through the regions indicated with arrows in Figure 1C and 1D. Rectangles outline areas magnified in panels C and D. Although concentration of ribosomes seems to be identical in *WT* and *Parp^CH1^* mutant, the total volume of cytoplasm is much smaller in *Parp^CH1^*. White bar shows cell size difference between *WT* and *Parp^CH^*. E–F. Sucrose density gradient analysis reveals the difference between ribosomal profiles in wild-type (*WT*), *Parg^27.1^* and *Parp^C03256^* mutants. E. A260 profiles of ribosome pools separated over sucrose density gradients. Positions of fractions corresponding to 40S and 60S subunits, 80S ribosomes and polysomes are indicated. F. Total proteins were extracted from corresponding fractions after sucrose density gradients (which are shown on panel E) and subjected to Western blot analysis using antibody against RPS6 protein, which belongs to the 40S ribosomal subunit. In wild-type samples, RPS6 protein labels fraction 15 (40S subunit itself), fractions 7–11 (mono-ribosome) and fractions 1–5 (polysomes). No polysomes were detected in either *Parg^27.1^* or *Parp^C03256^* mutants. Total level of mature mono-ribosomes is significantly decreased in *Parg^27.1^* mutants, although, total level of RNA (E, fractions 13–19) is much higher than in *WT*. Although antibody against ribosomal protein reveals ribosomal-related particles in fractions 7–15 in *Parp^C03256^* mutant, those particles could not be separated by sucrose gradient (E shows no picks). Last observation suggests incomplete processing or misfolding of ribosomes in *Parp^C03256^*. G. Detection of mRNA in Polysome Fractions. Quantitative real-time RT-PCR was used to analyze the amount of mRNA translated after disrupting PARP1 activity to measure functional ribosome complex formation. mRNA was isolated from 3rd instar larvae before and after polysome fractionation. Polysome fractions from each sample were combined together after isolating mRNA. Each dataset was normalized using Tubulin. The chart shows values obtained after normalizing each value generated before fractionation to values after polysome fractionation. Bars on the chart represent two independent experiments.

To examine the role of poly(ADP-ribosyl)ation in ribosomal production, we first compared the concentration of ribosomal particles in wild-type and *Parp* null mutant second-instar larvae using TEM analysis. We dissected larval midintestine from the same developmental stages of control and mutant animals, prepared ultra-thin sections from the same areas of midintestines (Figure 1C and 1D), and subjected these samples to EM analysis. Concentration of ribosomal particles was quantified using at least 5 sections for each sample. Although no difference in ribosome concentration was detected (compare Figure 6A and 6C and Figure 6B and 6D), we found a significant difference in total volume of cytoplasm between *Parp* null mutants and the wild-type. Cells of midintestine walls in *Parp* null mutant were, on average, twice as small as compared to those of the wild-type (Figure 6A and 6B, Figure S6) such phenotypes are typical of mutations within ribosome biogenesis pathways [6], [7].

We next compared ribosomal-polysomal profiles of wild-type and mutants within the pADPr turnover pathway using sucrose density gradient separation [6]. *Parp^CH1^* mutants arrest early in development and show a phenotype similar to small second-instar larvae which limited their use in sucrose density analysis. To analyze the effect of disrupting PARP1 activity on ribosomal assembly, we used *Parg^27.1^* and *Parp^C03256^* mutants that survive up to late pupae. Both mutants demonstrated an absence of polysomes and abnormal quantities of mature ribosomal subunits 40S, 60S, and mono-ribosomes 80S (Figure 6E and 6F). Moreover, both mutants show a marked decline in mRNA quantity within polysomal fractions (Figure 6G), suggesting problems with mRNAs translation. Taken together with the accumulation of uncleaved rRNA intermediates (Figure 5B), these last findings suggest the presence of a significant number of aberrant misfolded and unprocessed ribosomes in the cytoplasm of *Parg^27.1^* and *Parp^C03256^* cells.

## Discussion

Although substantial amount of PARP1 localizes in the nucleolus, prior to our study, very little was known about the function of this important protein in the nucleolus of *Drosophila*. We demonstrate that PARP1 activity is essential for the maintenance of *Drosophila* nucleolar structure and function, particularly for ribosome biogenesis. A number of nucleolar factors including Fibrillarin, AJ1, and CC01311 that co-localize in wild-type nucleolus, were observed to localize completely independent from one another when PARP1 function was disrupted. This suggests that the product of PARP1 enzymatic reaction, pADPr, may serve as a matrix for binding these nucleolar proteins and keeping them together in proximity to precursor rRNA. Our experiments with mutated PARP1 antagonist, PARG, identified a selected group of nucleolar proteins, including Fibrillarin, AJ1, Nucleolin, and Nucleophosmin, which were targeted to a specific location inside the nucleolus by PARP1 enzymatic reaction, apparently by binding of these proteins through attachment to pADPr matrix. Interestingly, although we observed a dramatic accumulation of 47S and 36S rRNA transcripts in the absence of a functional PARP1 activity, the level of 18S product was similar in both PARP1 wild-type and mutants. The accumulation of 47S and 36S rRNA transcripts can be attributed to either the upregulation of transcriptional activity in PARP1 mutants or defect in rRNA processing machinery. However, based on the dislocation of nucleolar proteins required for rRNA processing in PARP1 mutants, we believe that this accumulation is likely caused by the absence of a functional rRNA processing complex in PARP1 mutants. Furthermore, inhibiting PARP1 activity also lead to a significant reduction in the levels of ribosomes, suggesting that PARP1 activity is required for ribosome biogenesis. Taken together our findings suggest that by binding a specific set of nucleolar factors to pADPr, PARP1 likely determines the order of steps that occur during the process of ribosome biogenesis in the nucleolus.

The nucleolus is a site where the protein synthesizing machinery, the translational complex, is assembled. By virtue of this property, the nucleolus functions as a major regulator of cell growth in normal and cancer cells [43]. In addition to the proteins that make up the translational complex, the nucleolus also contains an array of proteins that function in cell cycle regulation, cell growth, and cell death induction upon exposure to DNA damaging agents [44], [45]. Findings reported here indicate that PARP1 activity is critical for nucleolar integrity and function. Recently published work by Guerrero and Maggert, support our findings that PARP1 activity is essential for the maintenance of nucleolar structure [46]. This results together with our data [23] highlights a role for PARP1 in nucleolar structure and maintenance. The research reported here extends beyond these analyses by examining PARP1 activity on the colocalization of nucleolar proteins, rRNA processing, and ribosome biogenesis. Since nucleolar function is essential during growth, this study suggests that PARP1 activity may play a central role in coordinating cell growth at the metabolic level. Here, we report our exciting observations that a novel PARP1 activity controls localization of critical components of ribosomal biogenesis within the nucleoli and therefore PARP1 is a critical regulator of ribosome production.

**Figure 7:**
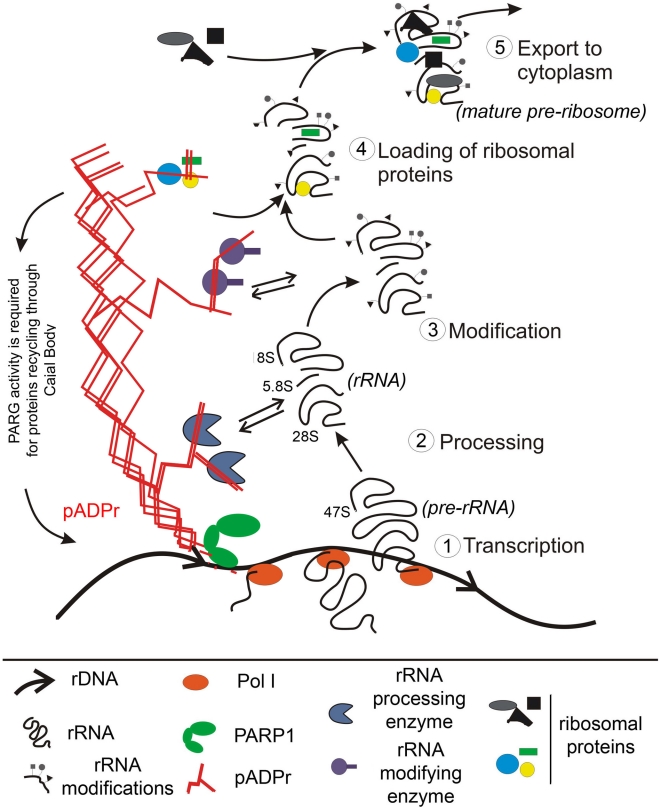
Nuclear PARP1 facilitates ribosomal biogenesis: a model. PARP1 protein becomes automodified upon each act of transcriptional start within rDNA gene and serves as a chaperoning machine during whole cycle of ribosome maturation in nucleolus. The dynamic Poly(ADP-ribose) tree forms a network, which organizes specific nucleolar microenvironment, brings a subset of nucleolar protein (such as Fibrillarin and AJ1) to the proximity of precursor rRNA, and coordinates the order of events of rRNA processing, modification, and loading of subsets of ribosomal proteins. Depletion of PARP1 protein leads to removal of pADPr-binding proteins from nucleoli, which disrupts processing, modification and folding of ribosomal RNA. PARG protein is required to 1) restart the system and 2) recycle protein components after completion of one cycle of ribosomal subunit synthesis.

Transcription of ribosomal DNA in nucleoli is performed specifically by the polymerase I machinery [47]. Although this transcriptional apparatus is very different from Pol II, the presence of poly(ADP-ribose) in nucleoli suggests that transcriptional start by Pol I involves PARP1 activation as it occurs with Pol II-dependent transcription [24], [48], [49]. By summarizing our data, we could propose that upon activation of rRNA synthesis, simultaneous activation of PARP1 leads to synthesis of an equal amount of pADPr, which “attracts” proteins required for rRNA processing, modification, and loading of an initial set of ribosomal proteins. PARP1 then coordinates the steps of ribosomal maturation and protects immature ribosomes from interacting with other groups of proteins that should be loaded last (Figure 7, Figures S7 and S8). To produce poly(ADP-ribose) and regulate production of ribosomes, PARP1 utilizes a pool of NAD which is linked to energy status of the cells. Therefore, our proposed model provides a new insight into the connection between the status of metabolism of an organism and translation and cell growth. Specifically, any event leading to a decrease of NAD level in a cell should slow down all PARP1 dependent processes in ribosome biogenesis and, therefore, change the rate of translational apparatus assembly.

While our results establish a direct connection between PARP1 and ribosome biogenesis, our findings do not exclude the possibility that PARP1 accumulation has other additional functions inside the nucleolus. One such function could involve protecting genomic stability of tandemly organized clusters of ribosomal genes. The presence of tandem arrays creates a possibility of unequal crossover, as a consequence of partial loosening of rDNA, which could be crucial for viability [50]. One of the first functions proposed for PARP1 protein upon its discovery was its involvement in DNA repair [51], [52]. Therefore, PARP1 may be a specific protector of rDNA that guards it against genetic instability by creating barrier between rDNA and enzymes involved in homology repair. Alternatively, the presence of negatively charged pADPr may create a microenvironment which blocks homologue recombination within tandem arrays and therefore protects these arrays from unequal crossover.

## Materials and Methods

### *Drosophila* strains and genetics

Flies were cultured on standard cornmeal-molasses-agar media at 22°C, unless otherwise indicated. The fly stocks were generated by the standard genetic methods or obtained from the Bloomington *Drosophila* Stock Center and the Exelixis Collection at the Harvard Medical School, except as indicated. Genetic markers are described in Flybase [53]. CC01311 GFP-trap stock was obtained from the A. Spradling Lab [33]. The *Parp^C03265^* strains were generated in a single pBac-element mutagenesis screen [54]. *Parg^27.1^* [19] and *Parp^CH1^* [23] mutants were previously described. pP{w1, UAS::PARG-EGFP}, called UAS:: PARG-EGFP, has also been previously described [21]. pP{w1, UAS::PARP1-DsRed}, called UAS::PARP1-DsRed, was described [23]. The following GAL4 driver strains were used: arm::GAL4 (Bloomington stock no. 1560), da::GAL4 (gift of A. Veraksa), and 69B-GAL4 [55]. Balancer chromosomes carrying Kr::GFP, i.e., TM3, Sb, P{w^+^, Kr-GFP} and FM7i, P{w1, Kr-GFP} [56], were used to identify heterozygous and homozygous *Parp^CH1^*, *Parp^C03265^* and *Parg^27.1^*.

### Construction of transgenic *Drosophila*

To construct the anti-Parp RNAi transgene we cloned an 1839-bp fragment of Parp-e cDNA (from GM10715 clone) in direct and inverted orientation within the pUASt vector. As a spacer between inverted repeats we used a 720-bp fragment of EGFP sequence (Figure S3). Transformation was as described [57], with modifications [58].

### Western blot

Protein extracts were separated on a 4–12% gel (Invitrogen), transferred onto a nitrocellulose membrane and detected using Amersham/GE Healthcare (#RPN2106) kit, according to manufacturer's instructions. The following primary antibodies were used: anti-Fibrillarin (rabbit, 1∶4000, gift form Dr. J. Gall), anti-Nucleolin (rabbit, 1∶4000), nucleolar AJ1 antibody (rabbit, 1∶1000, gift form Dr. J. Gall), anti-Nucleophosmin (rabbit, 1∶1000), anti-dNop5 (rabbit, 1∶2000, gift form Dr. G. Vorbruggen), anti-GFP (rabbit, Torrey Pines Biolabs, #TP401, 1∶1000), anti-CKIIα (rabbit, 1∶150, Stressgen, # KAP-ST010), anti-DLG (rabbit, 1∶5000, gift from Dr, F. Roegiers) and anti-RPS6 (mouse mAb, 1∶1000, Cell Signalling #2317).

### Electron microscopy

Electron microscopy was performed essentially as described [20].

### Immunoprecipitation

Immunoprecipitation experiments were performed as described [20], with little modifications. Briefly, 30 ul of Protein-G Sepharose 4B were added to the protein lysates and incubated overnight at 4°C with rotation. Beads were washed 4 times for 5 min in 1 ml of lysis buffer. Bound proteins were eluted using 60 ul of 1× Laemli with heating at 95°C for 5 min. The following antibodies were used for immunoprecipitation: anti-pADPr (Mouse mAb, H10 1∶20, Tulip, #1020).

### Northern blot

Total RNA was isolated using Trizol reagent (Gibco BRL), precipitated twice with 3 M LiCl, treated with Amplification Grade Dnase I (Gibco BRL). The following primers were used to produce ITS probe detecting intermediates of pre-rRNA: ITSf (5′-ataacaaaatgattccatgg-3′) and ITSr (5′-aaaaatacaccattttactgg-3′); for 18S rRNA probe: 18Sf (5′-aaaagtgaaaccgcaaaagg-3′) and 18Sr (5′-taatgatccttccccgcagg-3′). For Northern blot analysis, at least of 2.5 ug of total nuclear RNA from third instar larvae was used per lane. A *Tubulin* probe was used as a loading control.

### Sucrose density gradient analysis

Analysis of ribosomes by sucrose density gradient centrifugation was carried out as described [6], with modifications. Briefly, 3^rd^ instar larvae were picked, washed 2× in distilled water, followed by 20 min incubation at room temperature in 200 ul of Buffer A (20 mM HEPES [pH 7.5], 10 mM KCl, 2.5 mM MgCl_2_, 1 mM EGTA, 100 ug/ml cycloheximide, 1 mM DTT). 450 ul of Buffer A was then added, and larvae were then lysed on ice and centrifuged for 5 min at 10,000 rpm at 4°C. The absorbance of the supernatant was measured at 260 nm and 400 ug of each sample was carefully loaded onto a 10.5 ml 10–55% sucrose gradient in Buffer A without cycloheximide and DTT, centrifuged for 8 hrs at 27,000 rpm in a SW41 rotor. 1 ml fractions were collected using a Foxy Jr gradient collector (ISCO) with a UV detection system for recording profile. 25% of selected fractions were TCA-precipitated and analyzed by Western blot for the presence of S6 ribosomal protein (Cell Signaling). 380 ul of the remaining samples was used for RNA isolation. 30 ul of 10% SDS/Tris (pH 7.5) and 1 ml of combined phenol∶Chloroform∶Isoamyl Alcohol solution (invitrogen cat # 15593-031) was added to each sample and heated at 65°C for 2 min. Samples were centrifuged and the supernatant transferred to a new tube. Extraction was repeated again using the phenol∶Chloroform∶Isoamyl Alcohol solution, followed by transfer of the supernatant. 1/10 the volume of 3 M sodium acetate and 2× the volume of cold ethanol was added to each sample followed by incubation at −20°C for 20 min. Samples were centrifuged at 4°C for 4 min and the resulting pellet dissolved in appropriate volume of RNase free water to be used for qPCR analysis.

### Quantitative Reverse Transcription–PCR

10 ug of RNA was used for cDNA synthesis using the high capacity cDNA Reverse Transcription Kit (applied biosystems cat # 4368814). The following primer sequences: Tubulin: Forward (ccttcgtccactggtacgtt), Reverse (ggcgtgacgcttagtactcc); GAPDH: Forward (cgacaagttcgtgaagctga), Reverse (attctaccgcgccctaatct); Act5C: Forward (gtgcccatctacgagggtta), Reverse (agggcaacatagcacagctt) were utilized with Power SYBER Green master mix (cat # 4367659) for PCR using the Applied Biosystems StepOnePlus detection system with the following cycling conditions: 95°C for 10 min, followed by 40 (2-step) cycles (95°C, 15 s; 60°C, 60 s).

### Probes for *In Situ* Hybridization

rDNA probes were generated by digesting full length *Drosophila* rDNA gene (18S, 5.8S, and 28S) containing intergenic sequences with the following enzymes: HaeIII, AluI, MspI, RsaI, and MseI (New England Biolabs). Labeled probes were generated using DIG DNA Labeling Kit (Roche; Cat # 11 175 033 910) essentially as outlined by manufacturer.

### Fluorescence *In Situ* Hybridization (FISH)

FISH protocol was carried out as described [59], with modifications. Salivary glands were dissected from 3^rd^ instar larvae and fixed in for 10 min at room temperature (RT) in 4% formaldehyde in buffer A (15 mM PIPES, 80 mM KCl, 20 mM NaCl, 2 mM EDTA, 0.5 mM EGTA, 0.5 mM spermidine, 0.15 mM spermine, 1 mM DTT) prewarmed to 37°C. After fixing, salivary glands were washed three times (5 min each) in 2× SSCT (SSC in 0.1% Tween) followed by successive incubation at RT for 10 min in 20% formamide in 2× SSCT, 40% formamide in 2× SSCT, 50% formamide in 2× SSCT. Salivary glands were again incubated in fresh 50% formamide in 2× SSCT for 30 min at 37°C. The solution was aspirated and salivary glands were incubated with DNA probes diluted in 40 ul of hybridization solution (50% formamide, 3× SSCT, 20% dextran sulfate, 0.25% Tween). Probes and salivary glands were denatured (91°C for 2 min) together in a thermal cycler. Hybridization was carried out at 37°C for 24 hrs. Following hybridization, salivary glands were washed three times for 20 min each in 50% formamide in 2× SSCT at 37°C, and a wash in 25% formamide in 2× SSCT at RT. Salivary glands were washed again three times in 2× SSCT and 1× PBS/Tween at RT for 5 min each. Following washes, immunostaining protocol for Fibrillarin (outlined below) was carried out, starting by blocking with 10% BSA solution. The DIG labeled probes and Fibrillarin were detected by staining with rhodamine-conjugated anti-digoxigenin F(ab) fragments (Boehringer Mannheim) and Goat Anti-Mouse Alexa 488 diluted in 1× PBS/Tween at RT for 2 hrs. Salivary glands were washed with 1× PBS/Tween at RT twice, 20 min each time followed by incubation with DNA binding dye, Drag5, for 1 hr at RT.

### Immunostaining of salivary gland

Salivary glands were dissected out in Grace's medium brought to room temperature. Salivary glands were then moved directly into fixative solution of 2% formaldehyde in PBS containing 1% Triton X-100 (PBT) (in 1.5 mL Eppendorf tube) and rotated at room temperature for 30 min. After washing twice for 5 min each in PBT, blocking solution of PBT containing 10% bovine serum albumin (10% BSA) was applied to salivary glands and rotated at room temperature for 1 hr. After blocking in 10% BSA solution, salivary glands were washed in PBT containing 1% bovine serum albumin (1% BSA) for 5 min. Primary antibodies were then applied to salivary glands. Mouse Anti-Nop1p Fibrillarin (Corning) at a dilution of 1∶200, Rabbit Anti-Nucleolin (Abcam) at a dilution of 1∶200, Rabbit Anti-Fibrillarin (Abcam) at a dilution of 1∶500, Rabbit Anti-dNop5 (1∶400), Rabbit nucleolar AJ1 antibody (1∶400) and Rabbit Anti-GFP (1∶500) were applied to respective samples. Salivary glands were incubated in primary antibody overnight at 4 degrees on a rotator. After that, samples were washed in 1% BSA solution three times for 10 min each. Salivary glands were incubated with appropriate secondary antibody at room temperature on rotator for 2 hrs, and Goat Anti-Mouse Alexa 488, Goat Anti-Rabbit Alexa 568, Goat Anti-Rabbit Alexa 488 and Alexa 633 (from Molecular Probes) at a dilution of 1∶400 were applied. Next, samples were washed twice in PBT buffer for 5 min and then subjected to chromatin staining using Draq5 (Biostatus) at a dilution 1∶500 in PBT buffer for 1 hr at room temperature on rotator or Oligreen (Invitrogen) at a dilution of 1∶10,000 in PBT buffer solution for 10 min at room temperature. Salivary glands stained with Oligreen were then washed twice for 5 min in PBT buffer solution and fixed to microscope slide. Images were obtained using the Leica (DM-IRB) Confocal System.

## Supporting Information

Figure S1 Poly(ADP-ribose) accumulates in nucleoli. The dissected salivary glands from wild-type *Drosophila* were fixed and partially squashed on a slide, followed by immunostaining with anti-pADPr antibody (red) and with the DNA binding dye DAPI (blue). The arrow indicates the position of the nucleolus.
(TIF)

Figure S2 Nucleolar proteins, Fibrillarin and Aj1, are translocated to cytoplasm in *Parp* mutant cells. The total protein extracts from cytoplasmic and nuclear fractions of wild-type (WT) and Parp mutant second instar larvae were tested using Western blot. To detect protein on Western blots, the following antibodies were used: rabbit anti-Fibrillarin, rabbit anti-Aj1 and mouse anti-Actin, rabbit anti-H2A.
(TIF)

Figure S3 Structures of *UAS-Parp1-DsRed*, *Parpe-EGFP* and *Parp^RNAi^* transgenes. Nucleotide positions in Parpe cDNA are indicated. Functional domains are shown for Parp1-DsRed, Parpe-EGFP: Zn - Zn-finger; AM – automodification domain; PS – PARP signature (catalytic domain).
(TIF)

Figure S4 Casein kinase II α (CKIIα) protein (green) co-localizes with PARP1 protein (red) in wild-type nucleoli and antagonizing PARP1 in *Parg* mutants. DNA was detected by Draq5 dye (blue). N indicates nucleolus in one of wild-type nuclei. Arrow shows antagonistic localization of CKIIα and PARP1 in *Parg* mutant nucleolus.
(TIF)

Figure S5 Production of rRNA intermediates increases upon disruption of PARP1 activity. Northern blot analysis of rRNA intermediates. Disruption of PARP1 activity enhances the production of rRNA intermediates (right lane) compared to the wild-type (left lane), which has normal PARP1 activity. Labeled probe to *Drosophila* Tubulin mRNA was used as a loading control.
(TIF)

Figure S6 Mutating PARP1 affects cell growth. Confocal microscopy images of sections through midintestine of wild-type first and second instar larvae (top) and *Parp^CH1^* mutant second-instar larvae (bottom). Although the number of nuclei is identical in *WT* and *Parp^CH1^* mutant, the total volume of this organ is much smaller in *Parp^CH1^*.
(TIF)

Figure S7 Model: Mutating PARP1 protein disrupts ribosomal biogenesis. Depletion of PARP1 protein leads to removal of pADPr-binding proteins from nucleoli, which disrupts processing, modification and folding of ribosomal RNA. Therefore, multiple immature ribosomal complexes are accumulated.
(TIF)

Figure S8 Model: Mutating PARG protein disrupts ribosomal biogenesis. Depletion of PARG protein leads to arrest of PARP1 and pADPr-binding proteins in Cajal Bodies, which disrupts processing, modification and folding of ribosomal RNA. Therefore, multiple immature ribosomal complexes are exported to cytoplasm.
(TIF)

## References

[1] Boisvert FM, van Koningsbruggen S, Navascues J, Lamond AI (2007). The multifunctional nucleolus.. Nat Rev Mol Cell Biol.

[2] Frank DJ, Edgar BA, Roth MB (2002). The Drosophila melanogaster gene brain tumor negatively regulates cell growth and ribosomal RNA synthesis.. Development.

[3] Roger B, Moisand A, Amalric F, Bouvet P (2002). Repression of RNA polymerase I transcription by nucleolin is independent of the RNA sequence that is transcribed.. J Biol Chem.

[4] Tautz D, Hancock JM, Webb DA, Tautz C, Dover GA (1988). Complete sequences of the rRNA genes of Drosophila melanogaster.. Mol Biol Evol.

[5] Prieto JL, McStay B (2005). Nucleolar biogenesis: the first small steps.. Biochem Soc Trans.

[6] Milkereit P, Gadal O, Podtelejnikov A, Trumtel S, Gas N (2001). Maturation and intranuclear transport of pre-ribosomes requires Noc proteins.. Cell.

[7] Belin S, Beghin A, Solano-Gonzalez E, Bezin L, Brunet-Manquat S (2009). Dysregulation of ribosome biogenesis and translational capacity is associated with tumor progression of human breast cancer cells.. PLoS ONE.

[8] D'Amours D, Desnoyers S, D'Silva I, Poirier GG (1999). Poly(ADP-ribosyl)ation reactions in the regulation of nuclear functions.. Biochem J.

[9] Aubin RJ, Frechette A, de Murcia G, Mandel P, Lord A (1983). Correlation between endogenous nucleosomal hyper(ADP-ribosyl)ation of histone H1 and the induction of chromatin relaxation.. EMBO J.

[10] Boulikas T (1990). Poly(ADP-ribosylated) histones in chromatin replication.. J Biol Chem.

[11] Ji Y, Tulin AV (2010). The roles of PARP1 in gene control and cell differentiation.. Curr Opin Genet Dev.

[12] Krupitza G, Cerutti P (1989). Poly(ADP-ribosylation) of histones in intact human keratinocytes.. Biochemistry.

[13] Schreiber V, Dantzer F, Ame JC, de Murcia G (2006). Poly(ADP-ribose): novel functions for an old molecule.. Nat Rev Mol Cell Biol.

[14] Mendoza-Alvarez H, Alvarez-Gonzalez R (2001). Regulation of p53 sequence-specific DNA-binding by covalent poly(ADP-ribosyl)ation.. J Biol Chem.

[15] Malanga M, Pleschke JM, Kleczkowska HE, Althaus FR (1998). Poly(ADP-ribose) binds to specific domains of p53 and alters its DNA binding functions.. J Biol Chem.

[16] Pleschke JM, Kleczkowska HE, Strohm M, Althaus FR (2000). Poly(ADP-ribose) binds to specific domains in DNA damage checkpoint proteins.. J Biol Chem.

[17] Reale A, Matteis GD, Galleazzi G, Zampieri M, Caiafa P (2005). Modulation of DNMT1 activity by ADP-ribose polymers.. Oncogene.

[18] Davidovic L, Vodenicharov M, Affar EB, Poirier GG (2001). Importance of poly(ADP-ribose) glycohydrolase in the control of poly(ADP-ribose) metabolism.. Exp Cell Res.

[19] Hanai S, Kanai M, Ohashi S, Okamoto K, Yamada M (2004). Loss of poly(ADP-ribose) glycohydrolase causes progressive neurodegeneration in Drosophila melanogaster.. Proc Natl Acad Sci U S A.

[20] Kotova E, Jarnik M, Tulin AV (2009). Poly (ADP-ribose) polymerase 1 is required for protein localization to Cajal body.. PLoS Genet.

[21] Tulin A, Naumova NM, Menon AK, Spradling AC (2006). Drosophila poly(ADP-ribose) glycohydrolase mediates chromatin structure and SIR2-dependent silencing.. Genetics.

[22] Mendoza-Alvarez H, Alvarez-Gonzalez R (1993). Poly(ADP-ribose) polymerase is a catalytic dimer and the automodification reaction is intermolecular.. J Biol Chem.

[23] Tulin A, Stewart D, Spradling AC (2002). The Drosophila heterochromatic gene encoding poly(ADP-ribose) polymerase (PARP) is required to modulate chromatin structure during development.. Genes Dev.

[24] Tulin A, Spradling A (2003). Chromatin loosening by poly(ADP)-ribose polymerase (PARP) at Drosophila puff loci.. Science.

[25] Desnoyers S, Kaufmann SH, Poirier GG (1996). Alteration of the nucleolar localization of poly(ADP-ribose) polymerase upon treatment with transcription inhibitors.. Exp Cell Res.

[26] Meder VS, Boeglin M, de Murcia G, Schreiber V (2005). PARP-1 and PARP-2 interact with nucleophosmin/B23 and accumulate in transcriptionally active nucleoli.. J Cell Sci.

[27] Chan PK (1992). Characterization and cellular localization of nucleophosmin/B23 in HeLa cells treated with selected cytotoxic agents (studies of B23-translocation mechanism).. Exp Cell Res.

[28] Leitinger N, Wesierska-Gadek J (1993). ADP-ribosylation of nucleolar proteins in HeLa tumor cells.. J Cell Biochem.

[29] Koyama Y, Katagiri S, Hanai S, Uchida K, Miwa M (1999). Poly(ADP-ribose) polymerase interacts with novel Drosophila ribosomal proteins, L22 and l23a, with unique histone-like amino-terminal extensions.. Gene.

[30] Pinnola A, Naumova N, Shah M, Tulin AV (2007). Nucleosomal core histones mediate dynamic regulation of poly(ADP-ribose) polymerase 1 protein binding to chromatin and induction of its enzymatic activity.. J Biol Chem.

[31] Tollervey D, Lehtonen H, Carmo-Fonseca M, Hurt EC (1991). The small nucleolar RNP protein NOP1 (fibrillarin) is required for pre-rRNA processing in yeast.. EMBO J.

[32] Kotova E, Jarnik M, Tulin AV (2010). Uncoupling of the transactivation and transrepression functions of PARP1 protein.. Proc Natl Acad Sci U S A.

[33] Buszczak M, Paterno S, Lighthouse D, Bachman J, Planck J (2007). The carnegie protein trap library: a versatile tool for Drosophila developmental studies.. Genetics.

[34] Vorbruggen G, Onel S, Jackle H (2000). Restricted expression and subnuclear localization of the Drosophila gene Dnop5, a member of the Nop/Sik family of the conserved rRNA processing factors.. Mech Dev.

[35] Ginisty H, Sicard H, Roger B, Bouvet P (1999). Structure and functions of nucleolin.. J Cell Sci.

[36] Bierhoff H, Dundr M, Michels AA, Grummt I (2008). Phosphorylation by casein kinase 2 facilitates rRNA gene transcription by promoting dissociation of TIF-IA from elongating RNA polymerase I.. Mol Cell Biol.

[37] Berges T, Petfalski E, Tollervey D, Hurt EC (1994). Synthetic lethality with fibrillarin identifies NOP77p, a nucleolar protein required for pre-rRNA processing and modification.. EMBO J.

[38] Jansen R, Tollervey D, Hurt EC (1993). A U3 snoRNP protein with homology to splicing factor PRP4 and G beta domains is required for ribosomal RNA processing.. EMBO J.

[39] Schimmang T, Tollervey D, Kern H, Frank R, Hurt EC (1989). A yeast nucleolar protein related to mammalian fibrillarin is associated with small nucleolar RNA and is essential for viability.. EMBO J.

[40] Perry RP (1976). Processing of RNA.. Annu Rev Biochem.

[41] Winicov I, Perry RP (1975). Enzymological aspects of processing of mammalian rRNA.. Brookhaven Symp Biol.

[42] Bailey-Serres J (1999). Selective translation of cytoplasmic mRNAs in plants.. Trends Plant Sci.

[43] Montanaro L, Trere D, Derenzini M (2008). Nucleolus, ribosomes, and cancer.. Am J Pathol.

[44] Olson MO, Hingorani K, Szebeni A (2002). Conventional and nonconventional roles of the nucleolus.. Int Rev Cytol.

[45] Rubbi CP, Milner J (2003). Disruption of the nucleolus mediates stabilization of p53 in response to DNA damage and other stresses.. EMBO J.

[46] Guerrero PA, Maggert KA (2011). The CCCTC-binding factor (CTCF) of Drosophila contributes to the regulation of the ribosomal DNA and nucleolar stability.. PLoS ONE.

[47] Mandal RK, Dawid IB (1981). The nucleotide sequence at the transcription termination site of ribosomal RNA in Drosophila melanogaster.. Nucleic Acids Res.

[48] Petesch SJ, Lis JT (2008). Rapid, transcription-independent loss of nucleosomes over a large chromatin domain at Hsp70 loci.. Cell.

[49] Tulin A, Chinenov Y, Spradling A (2003). Regulation of chromatin structure and gene activity by poly(ADP-ribose) polymerases.. Curr Top Dev Biol.

[50] Kobayashi T (2011). Regulation of ribosomal RNA gene copy number and its role in modulating genome integrity and evolutionary adaptability in yeast.. Cell Mol Life Sci.

[51] Skidmore CJ, Davies MI, Goodwin PM, Halldorsson H, Lewis PJ (1979). The involvement of poly(ADP-ribose) polymerase in the degradation of NAD caused by gamma-radiation and N-methyl-N-nitrosourea.. Eur J Biochem.

[52] Durkacz BW, Omidiji O, Gray DA, Shall S (1980). (ADP-ribose)n participates in DNA excision repair.. Nature.

[53] (1999). The FlyBase database of the Drosophila Genome Projects and community literature. The FlyBase Consortium.. Nucleic Acids Res.

[54] Artavanis-Tsakonas S (2004). Accessing the Exelixis collection.. Nat Genet.

[55] Manseau L, Baradaran A, Brower D, Budhu A, Elefant F (1997). GAL4 enhancer traps expressed in the embryo, larval brain, imaginal discs, and ovary of Drosophila.. Dev Dyn.

[56] Casso D, Ramirez-Weber F, Kornberg TB (2000). GFP-tagged balancer chromosomes for Drosophila melanogaster.. Mech Dev.

[57] Spradling AC, Rubin GM (1982). Transposition of cloned P elements into Drosophila germ line chromosomes.. Science.

[58] Prokhorova AV, Voloshina MA, Shostak NG, Barskii VE, Golubovskii MD (1994). [Preparation and primary genetic analysis of Drosophila melanogaster transformants line w'lz(b)/XXywf, containing mini-white genes, integrated in the genome during P-element-dependent transformation].. Genetika.

[59] Dernburg AF, Sedat JW, Hawley RS (1996). Direct evidence of a role for heterochromatin in meiotic chromosome segregation.. Cell.

