# Supplementary figures and images for: Poly(ADP-Ribose) Polymerase 1 (PARP-1) Regulates Ribosomal Biogenesis in Drosophila Nucleoli
Source: PLoS Genet. 2012 Jan 5;8(1):e1002442. doi: 10.1371/journal.pgen.1002442 (PMC3252306; doi:10.1371/journal.pgen.1002442)

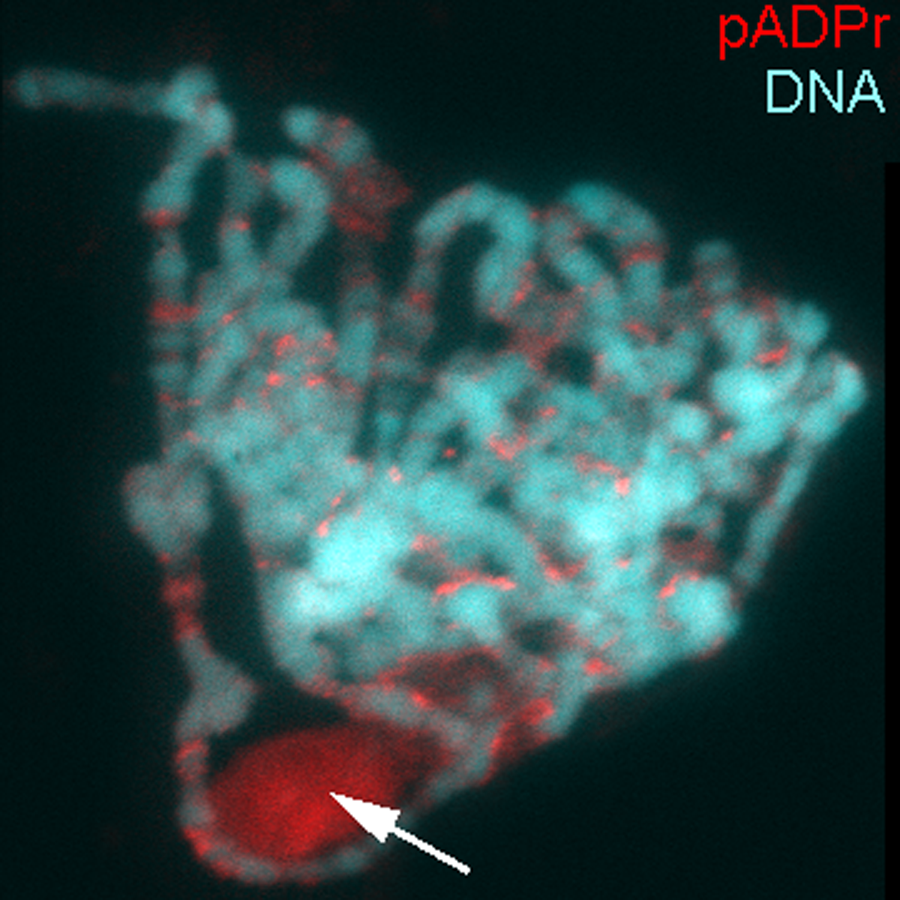

Supplement: Figure S1 — Poly(ADP-ribose) accumulates in nucleoli. The dissected salivary glands from wild-type Drosophila were fixed and partially squashed on a slide, followed by immunostaining with anti-pADPr antibody (red) and with the DNA binding dye DAPI (blue). The arrow indicates the position of the nucleolus. (TIF) [file pgen.1002442.s001.tif]

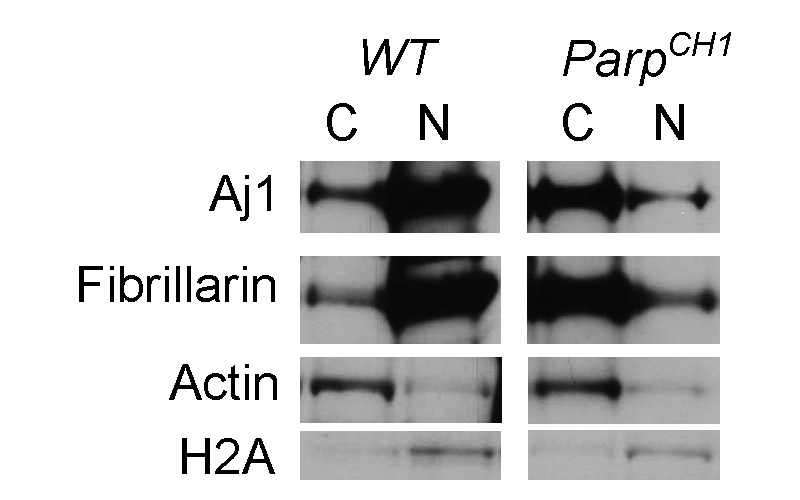

Supplement: Figure S2 — Nucleolar proteins, Fibrillarin and Aj1, are translocated to cytoplasm in Parp mutant cells. The total protein extracts from cytoplasmic and nuclear fractions of wild-type (WT) and Parp mutant second instar larvae were tested using Western blot. To detect protein on Western blots, the following antibodies were used: rabbit anti-Fibrillarin, rabbit anti-Aj1 and mouse anti-Actin, rabbit anti-H2A. (TIF) [file pgen.1002442.s002.tif]

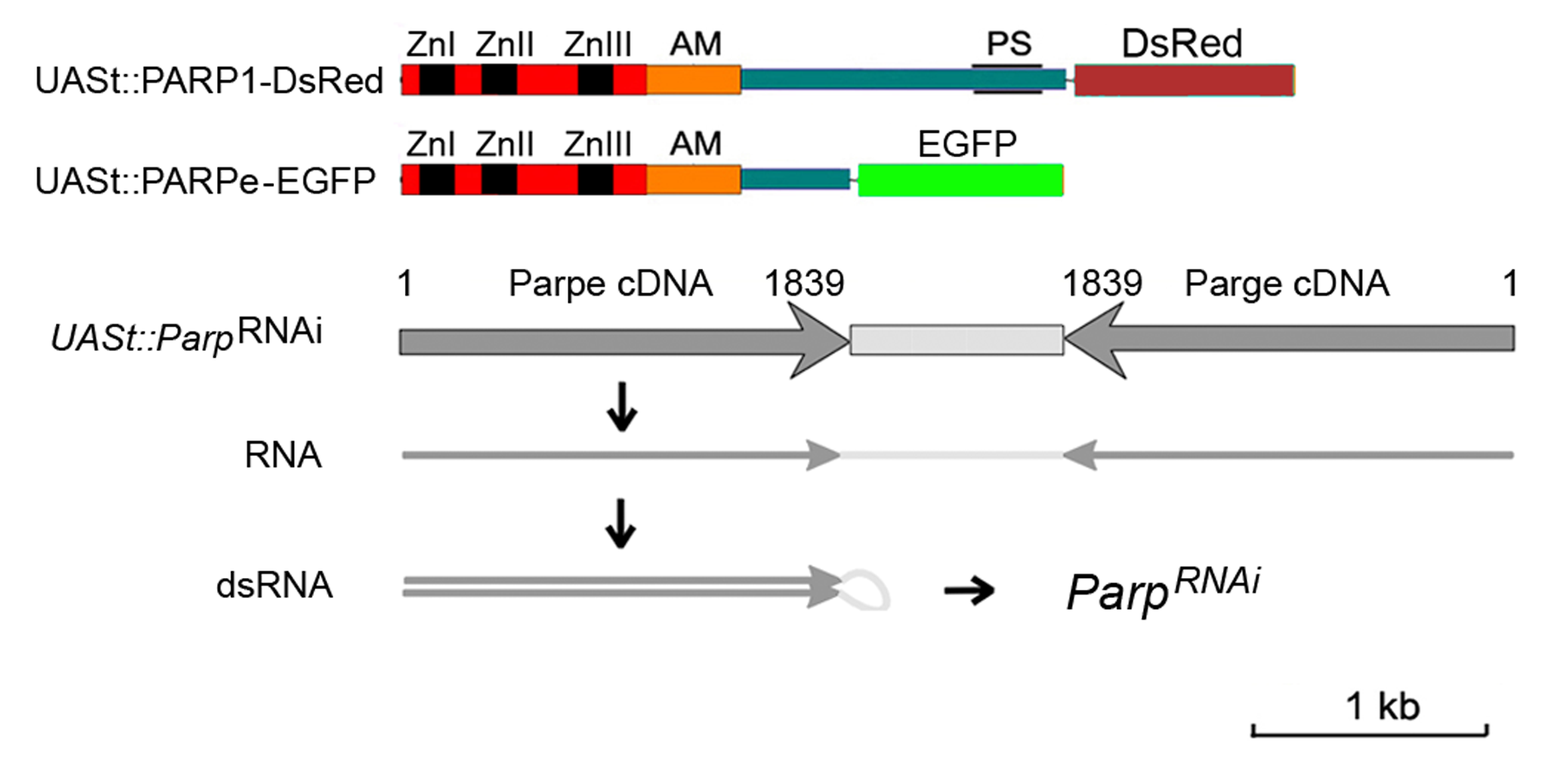

Supplement: Figure S3 — Structures of UAS-Parp1-DsRed, Parpe-EGFP and ParpRNAi transgenes. Nucleotide positions in Parpe cDNA are indicated. Functional domains are shown for Parp1-DsRed, Parpe-EGFP: Zn - Zn-finger; AM – automodification domain; PS – PARP signature (catalytic domain). (TIF) [file pgen.1002442.s003.tif]

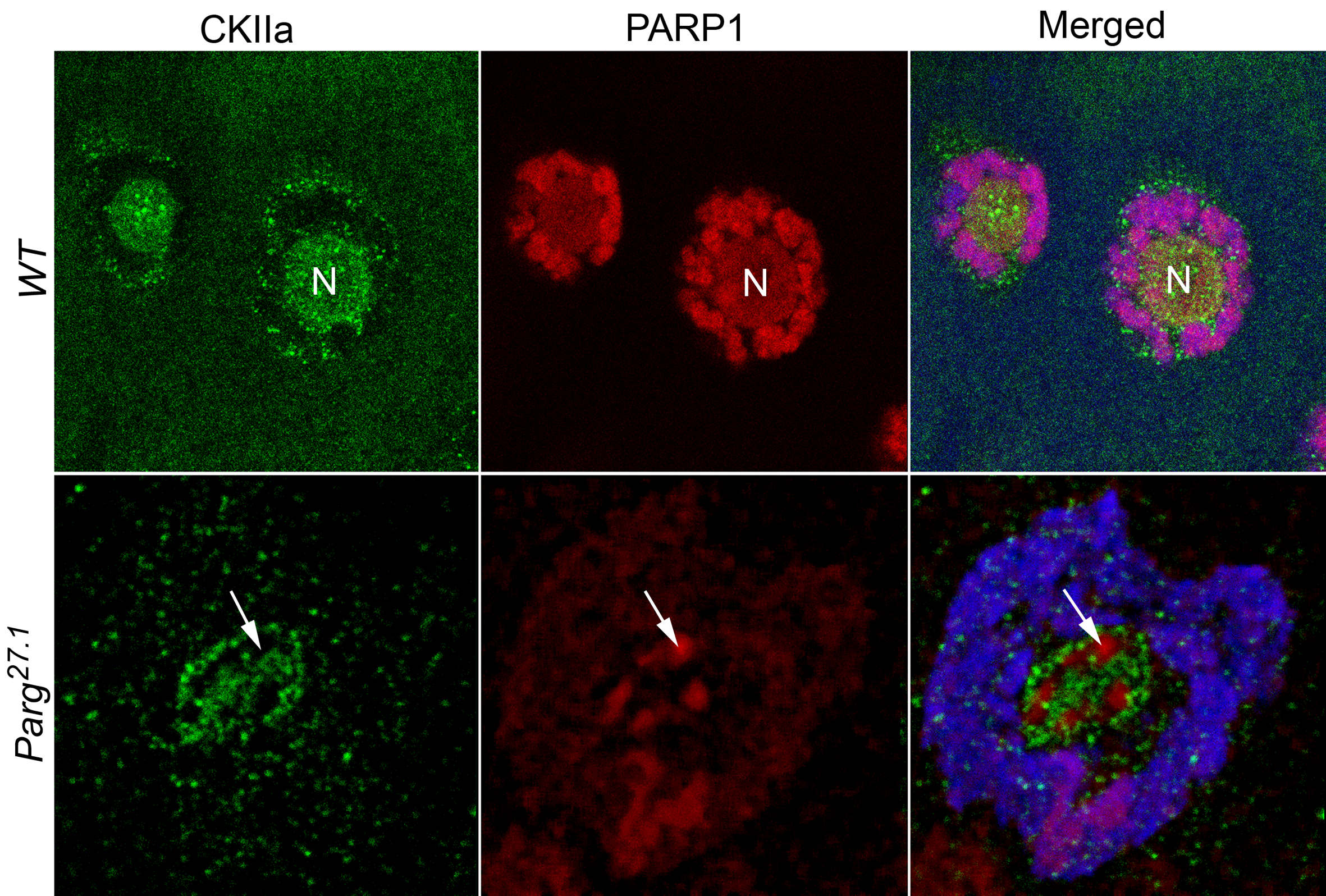

Supplement: Figure S4 — Casein kinase II α (CKIIα) protein (green) co-localizes with PARP1 protein (red) in wild-type nucleoli and antagonizing PARP1 in Parg mutants. DNA was detected by Draq5 dye (blue). N indicates nucleolus in one of wild-type nuclei. Arrow shows antagonistic localization of CKIIα and PARP1 in Parg mutant nucleolus. (TIF) [file pgen.1002442.s004.tif]

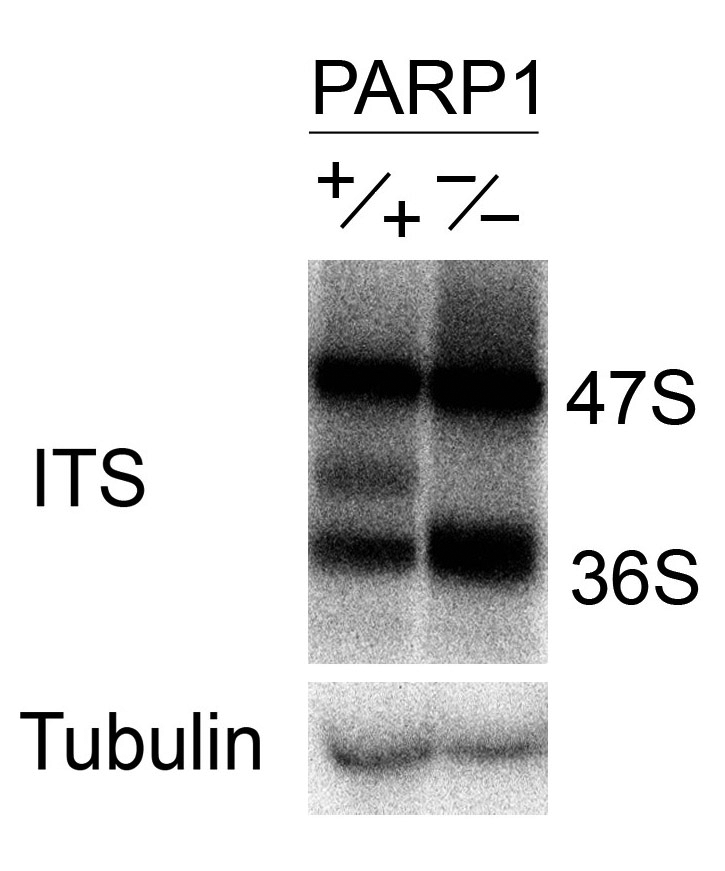

Supplement: Figure S5 — Production of rRNA intermediates increases upon disruption of PARP1 activity. Northern blot analysis of rRNA intermediates. Disruption of PARP1 activity enhances the production of rRNA intermediates (right lane) compared to the wild-type (left lane), which has normal PARP1 activity. Labeled probe to Drosophila Tubulin mRNA was used as a loading control. (TIF) [file pgen.1002442.s005.tif]

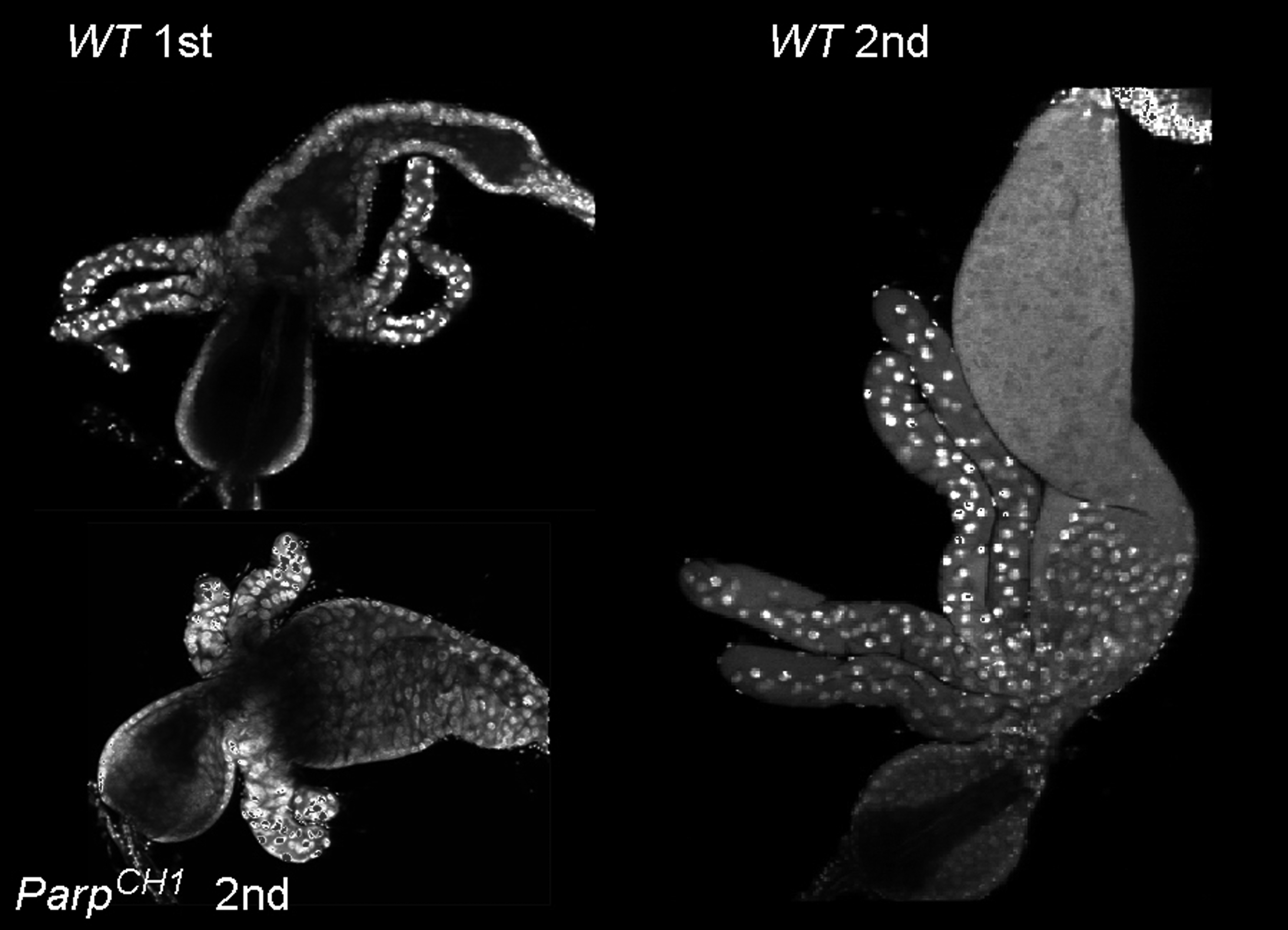

Supplement: Figure S6 — Mutating PARP1 affects cell growth. Confocal microscopy images of sections through midintestine of wild-type first and second instar larvae (top) and ParpCH1 mutant second-instar larvae (bottom). Although the number of nuclei is identical in WT and ParpCH1 mutant, the total volume of this organ is much smaller in ParpCH1. (TIF) [file pgen.1002442.s006.tif]

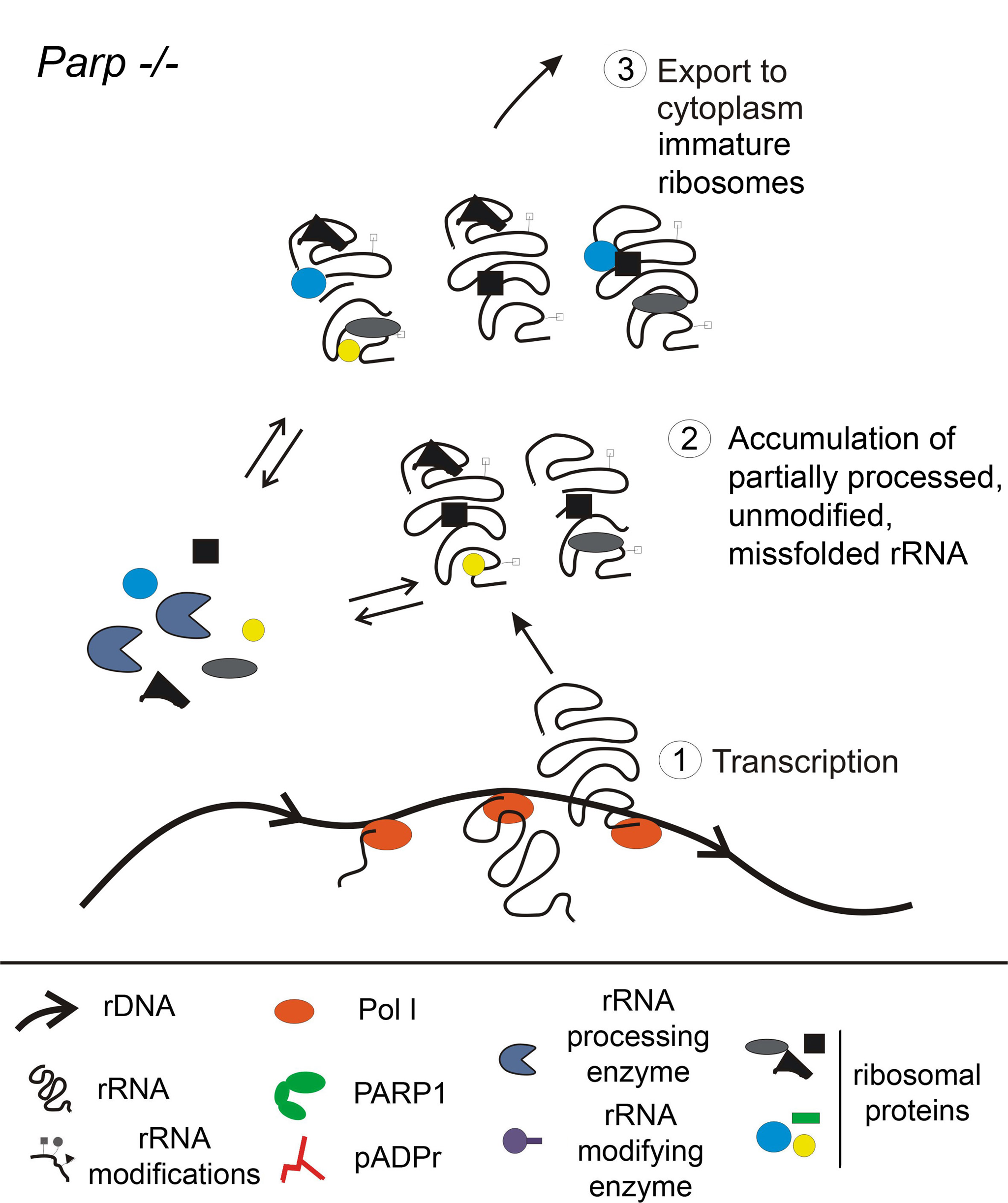

Supplement: Figure S7 — Model: Mutating PARP1 protein disrupts ribosomal biogenesis. Depletion of PARP1 protein leads to removal of pADPr-binding proteins from nucleoli, which disrupts processing, modification and folding of ribosomal RNA. Therefore, multiple immature ribosomal complexes are accumulated. (TIF) [file pgen.1002442.s007.tif]

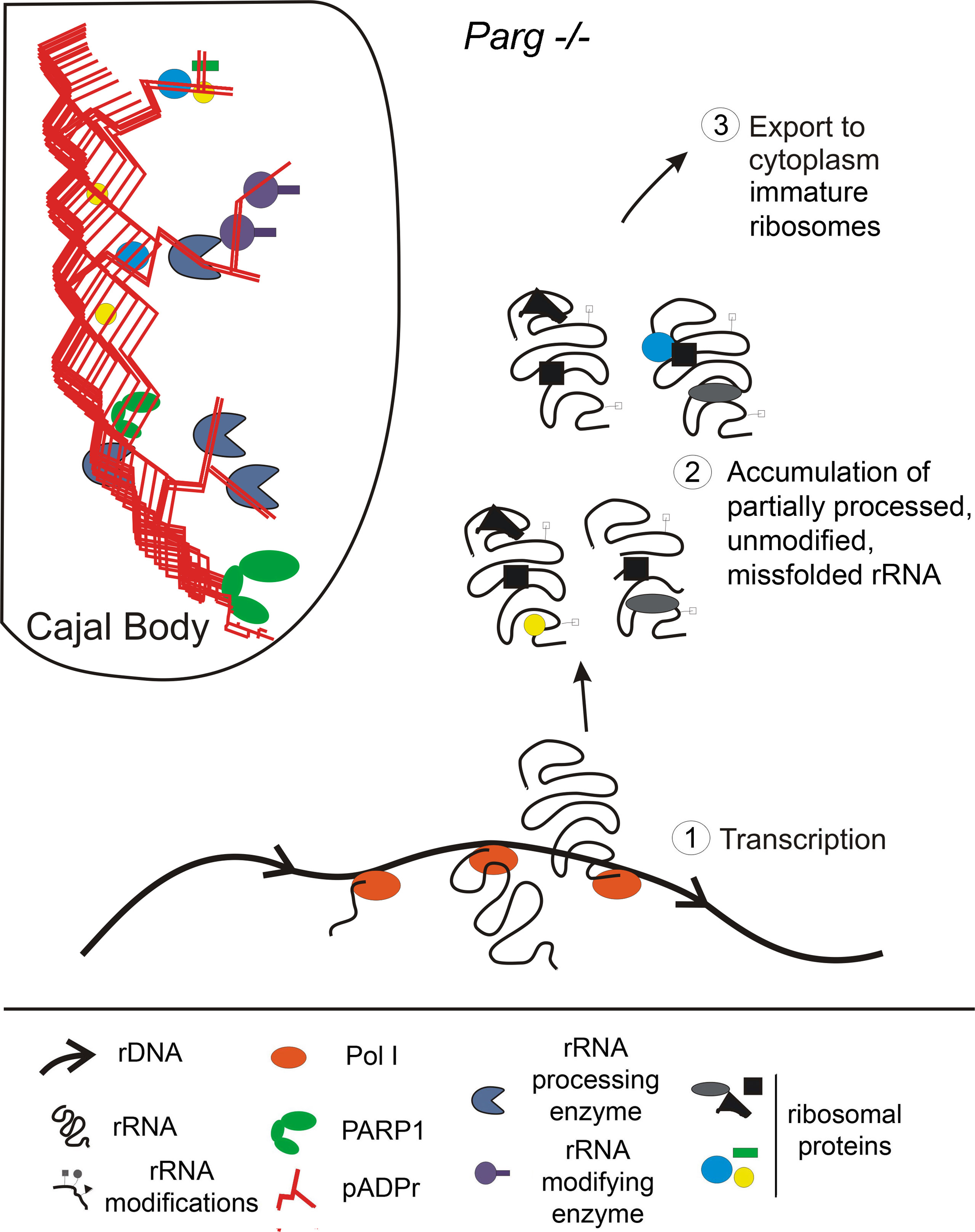

Supplement: Figure S8 — Model: Mutating PARG protein disrupts ribosomal biogenesis. Depletion of PARG protein leads to arrest of PARP1 and pADPr-binding proteins in Cajal Bodies, which disrupts processing, modification and folding of ribosomal RNA. Therefore, multiple immature ribosomal complexes are exported to cytoplasm. (TIF) [file pgen.1002442.s008.tif]
